# Extranodal extension of lymph node metastasis influences recurrence in prostate cancer: a systematic review and meta-analysis

**DOI:** 10.1038/s41598-017-02577-4

**Published:** 2017-05-24

**Authors:** Claudio Luchini, Achim Fleischmann, Joost L. Boormans, Matteo Fassan, Alessia Nottegar, Paola Lucato, Brendon Stubbs, Marco Solmi, Antonio Porcaro, Nicola Veronese, Matteo Brunelli, Aldo Scarpa, Liang Cheng

**Affiliations:** 1 Department of Diagnostics and Public Health, University and Hospital Trust of Verona, Verona, Italy; 2 ARC-NET Research Center, University and Hospital Trust of Verona, Verona, Italy; 3 Department of Pathology, Santa Chiara Hospital, Trento, Italy; 4 Institute of Pathology, University of Bern, CH-3010 Bern, Switzerland; 5 Department of Urology, Erasmus MC - Cancer Institute, Rotterdam, The Netherlands; 6 Department of Medicine, DIMED, University of Padua, Padua, Italy; 7 Health Service and Population Research Department, King’s College London, De Crespigny Park, London, SE5 8AF United Kingdom; 8 Department of Neuroscience, University of Padua, Padua, Italy; 9 Urologic Clinic, University and Hospital trust of Verona, Verona, Italy; 10 National Research Council, Neuroscience Institute, Aging Branch, Padova, Italy; 11 Institute for clinical Research and Education in Medicine (IREM), Padova, Italy; 12 Department of Pathology and Laboratory Medicine, Indiana University School of Medicine, Indianapolis, IN USA

## Abstract

The extranodal extension (ENE) of nodal metastasis involves the extension of neoplastic cells through the lymph node capsule into the perinodal adipose tissue. This morphological feature has recently been indicated as an important prognostic factor in various cancer types, but its role in prostate cancer is still unclear. We aimed to clarify it, performing the first meta-analysis on this issue, comparing prognostic parameters in surgically treated, node-positive prostate cancer patients with (ENE+) vs. without (ENE−) ENE. Data were summarized using risk ratios (RRs) for number of deaths/recurrences and hazard ratios (HRs), with 95% confidence intervals (CI), for the time-dependent risk related to ENE positivity. Six studies followed-up 1,113 patients with N1 prostate cancer (658 ENE+ vs. 455 ENE−) for a median of 83 months. The presence of ENE was associated with a significantly higher risk of biochemical recurrence (RR = 1.15; 95%CI: 1.03–1.28; I^2^ = 0%; HR = 1.40, 95%CI: 1.12–1.74; I^2^ = 0%) and “global” (biochemical recurrence and distant metastasis) recurrence (RR = 1.15; 95%CI: 1.04–1.28; I^2^ = 0%; HR = 1.41, 95%CI: 1.14–1.74; I^2^ = 0%). ENE emerged as a potential prognostic moderator, earmarking a subgroup of patients at higher risk of recurrence. It may be considered for the prognostic stratification of metastatic patients. New possible therapeutic approaches may explore more in depth this prognostic parameter.

## Introduction

**Figure 1:**
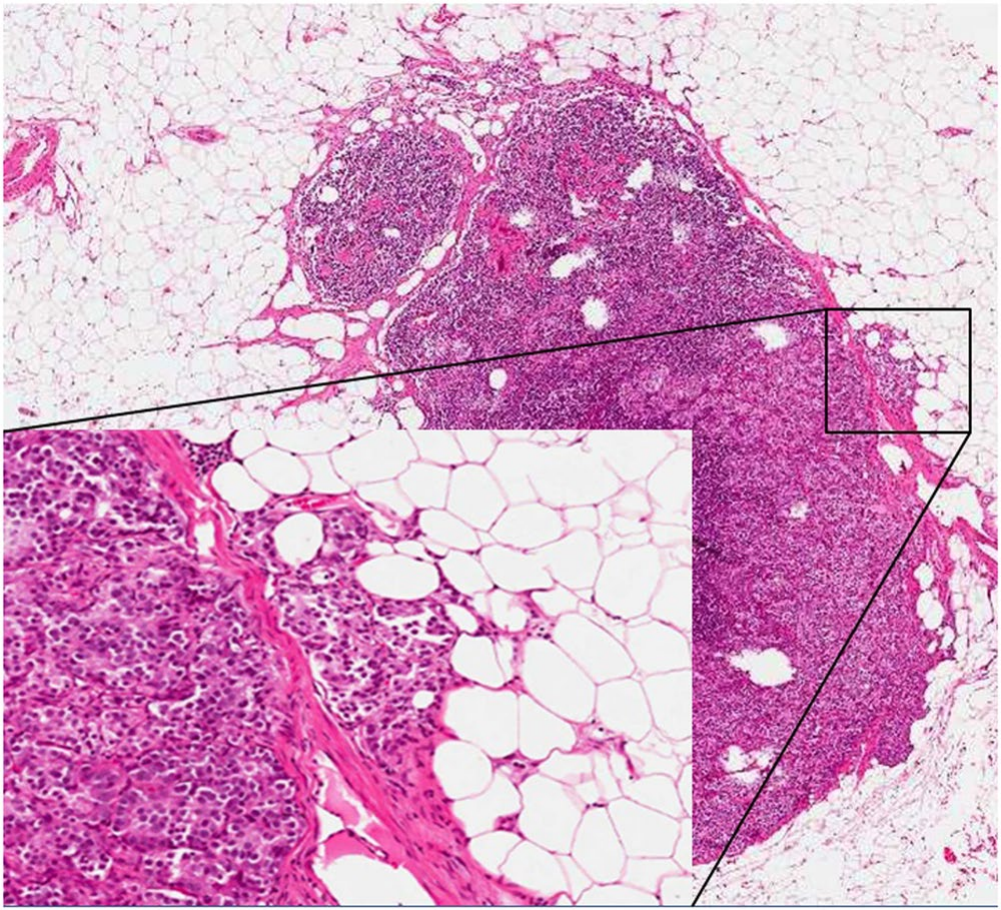
A classical example of extra-nodal extension of nodal metastasis of prostate cancer is here shown. Note the rupture of nodal capsule and the invasion by the metastatic cells of the peri-nodal adipose tissue (original magnification: 4× metastatic lymph node, 10× detail of the metastasis in the box).

Prostate cancer (PCa) is one of the most common cancers in men worldwide^1–4^, and deaths from prostate cancer are second only to those due to lung cancer^3^. The incidence and prevalence of prostate cancer are different in each area of the world, the highest being in North America and the lowest in Southern Asia^2^, but the worldwide incidence of PCa has grown substantially in recent years^1–4^. The prognosis of PCa patients depends mainly on the presence or absence of distant metastases^5^, and lymph node metastases are a particularly crucial prognostic factor^6^. Several researchers have analyzed the different morphological features of lymph node metastases in an effort to identify their most prognostically significant characteristics^7–15^. Some features (e.g. the involvement of multiple versus single lymph nodes, or nodal cancer volume) have revealed a strong prognostic value. Conventional staging for PCa does not differentiate between subgroups of node-positive disease; stratification is only based on the absence or presence of nodal metastases (N0 versus N1)^16^. There are also certain morphological aspects of lymph node metastases that have no clearly-established prognostic role yet, as different studies have generated different conclusions. One of the morphological features to consider is the presence of extranodal extension (ENE) of nodal metastases, which is indicated as the extension of metastatic cells beyond the nodal capsule into the perinodal soft tissue (Fig. 1). ENE has been recently indicated as a significant prognostic factor in many cancer types^17–25^, but its role in PCa is still unclear. For these reasons, and also because identifying generally-acceptable prognostic indicators is still a very important challenge in these times of personalized medicine, the aim of the present study was to establish the weight and determining the role of ENE on the prognosis of patients with N1 (nodal metastasis/metastases) PCa by performing the first meta-analysis on this argument.

## Results

### Search results

Altogether, 1051 non-duplicated articles were identified by our literature search. We have excluded 1024 articles after title/abstract review; the remaining 27 articles were retrieved for full text review. At last, after a complete analysis based on the criteria of eligibility, 6 studies resulted suitable for this meta-analysis (Supplementary Figure 1).

### Study and patient characteristics

The 6 meta-analyzed studies followed up 1,113 cases (658 ENE+ vs. 455 ENE−) over a median period of 83 months (range: 16–92) (Supplementary Table 1)^10–15^. The quality of the studies seemed to be good, and none of them showed a potentially high risk of bias (median NOS score = 8, range: 7–9; Supplementary Tables 1 and 2).

The mean age of patients was 65 ± 10 years; we do not find significant differences between ENE+ and ENE− patients in terms of mean age (Student’s t-test for independent samples, p = 0.88) or Gleason score (chi-square test, p = 0.21) (Supplementary Table 1).

### All-cause mortality (ACM), cancer-specific mortality (CSM) and risk of recurrence (ROR)

One study reported that 20/71 patients (=28.2%) with ENE+ vs. 5/31 (=16.1%) with ENE− died, meaning an increased risk of ACM in the former that was not statistically significant (RR = 1.75; 95%CI: 0.72–4.23; p = 0.22) (Table 1)^12^. Similarly, four studies reported that ENE + was not significantly associated with a higher risk of CSM (RR = 1.21; 95%CI: 0.98–1.50; p = 0.08; I^2^ = 0%)^10–13^.

**Table 1:** Pooled risk ratio estimates for overall and disease-free survival by presence or absence of extranodal extension.

Parameter	N. of studies	No. of events in ENE+	No. of ENE+	No. of events in ENE−	No. of ENE−	Risk ratio (95% CI)	P-value	Heterogeneity	Egger’s bias test; p-value	Trim and fill [trimmed]
ACM	1	20	71	5	31	1.75 (0.72–4.23)	0.22	—	—	—
CSM	4	97	312	51	198	1.21 (0.98–1.50)	0.08	I^2^ = 0	0.87; 0.30	1.18 (0.94–1.50)^1^
ROR	3	248	477	163	321	**1.15 (1.04–1.28)**	**0.008**	I^2^ = 0	0.29; 0.87	Unchanged

Abbreviations: ENE: extranodal extension; CI: confidence interval; ACM: all-cause mortality; CSM: cancer-specific mortality; ROR: risk of recurrence.

Bold values are significant results with p-values < 0.05.

**Figure 2:**
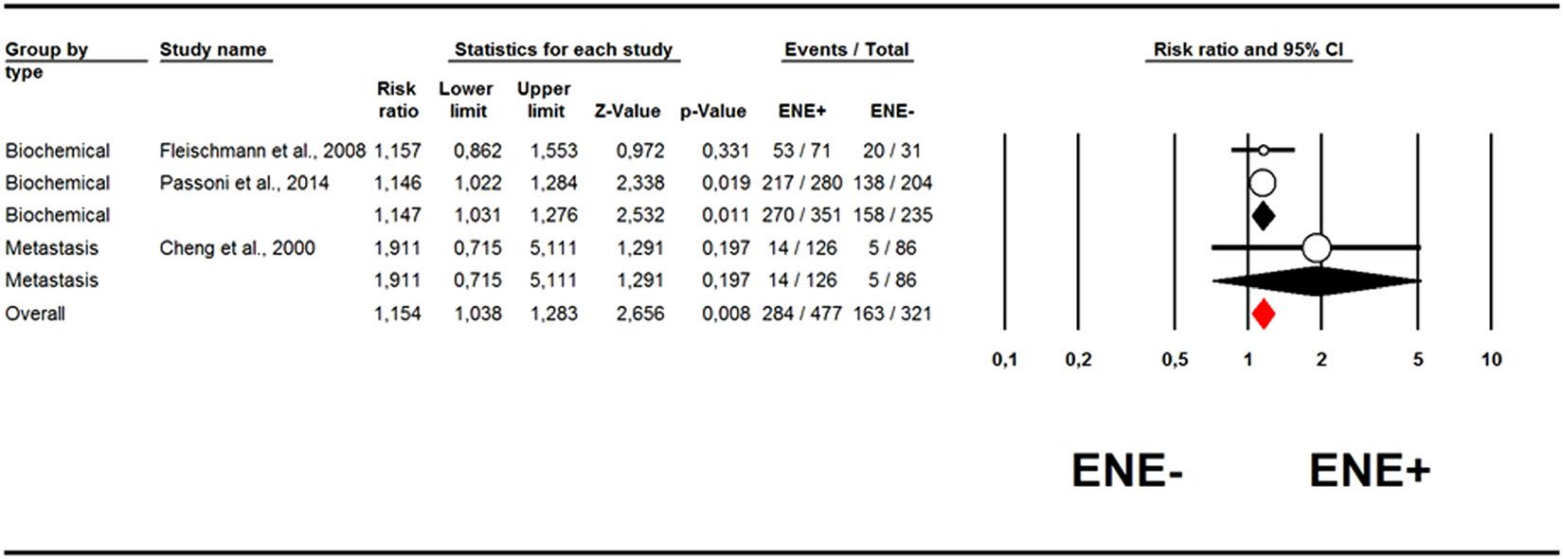
Forrest plot for relative risk of recurrence by extranodal extension status. First of all we present the data about risk of biochemical recurrence (papers of Fleischmann *et al*., and of Passoni *et al*.), then their meta-analyzed values (pooling data), then the data about risk of metastasis (Cheng *et al*.), its mean (only this study about the risk of metastasis, so we repeat this value), then the overall meta-analysis of these data (meta-analysis of all these papers about risk of recurrence – both biochemical and metastasis).

With regard to ROR, ENE+ status was associated with a higher risk of BCR (RR = 1.15; 95%CI: 1.03–1.28; p = 0.01; I^2^ = 0%) in two studies^12, 15^, while this association was not significant (RR = 1.91; 95%CI: 0.72–5.11; p = 0.20) in one study^11^ that considered metastasis as an indicator of recurrence (Fig. 2). Pooling data of all the three studies about ROR^11, 12, 15^, the global association with ENE was also significant (RR = 1.15; 95%CI: 1.04–1.28; p = 0.008; I^2^ = 0%, Table 1 and Fig. 2). The p for the interaction between BCR and solid metastasis was = 0.46, however, suggesting that the type of outcome was not a significant moderator of these findings.

No publication bias emerged for any of the outcomes considered (Table 1).

### Adjusted HRs on ACM, CSM and ROR

In secondary analyses, we analyzed whether using HRs (hazard ratios were adjusted for the maximum number of covariates available in each study) instead of RRs could affect our results. In the survival analyses, the median number of adjustments used was 1 (range: 0–8) (Supplementary Table 1).

**Figure 3:**
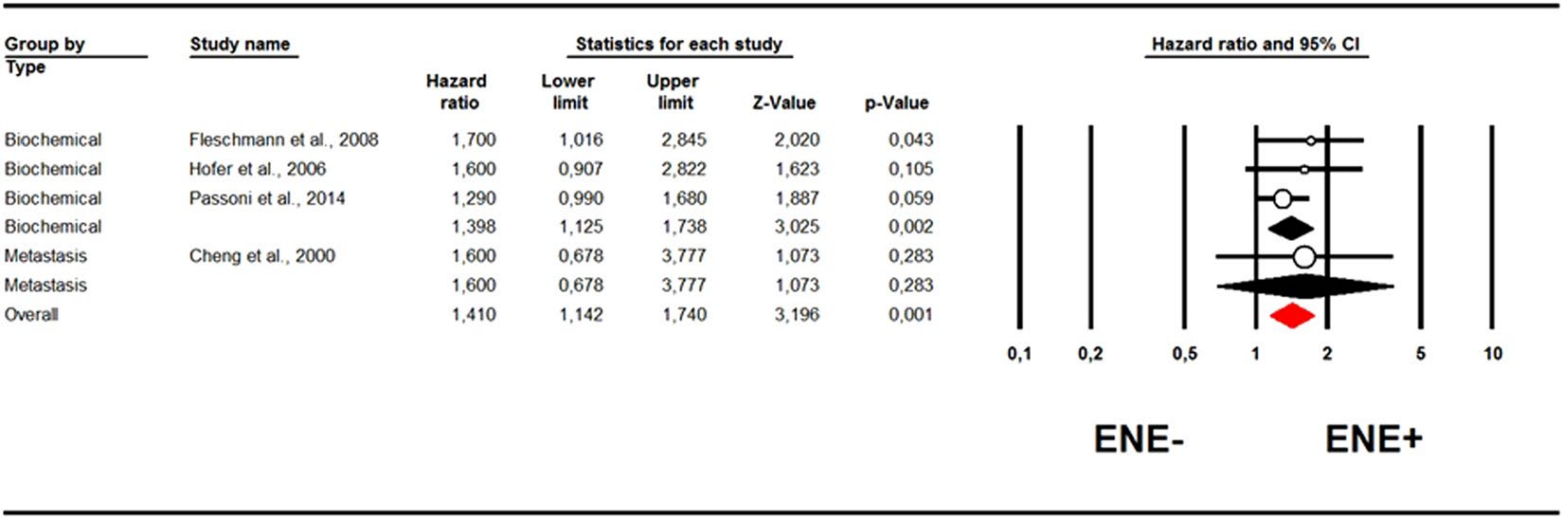
Forrest plot for hazard ratios (HRs) for recurrence (adjusted for potential confounders) by extranodal extension status. We present data in the same manner used for Fig. 2. The only difference is that there is an additional paper (Hofer *et al*.) for the risk biochemical recurrence.

Table 2 shows the adjusted HRs by ENE status. ENE+ status was not associated with a significantly worse prognosis than ENE− status when ACM (1 study; HR = 1.50, 95%CI: 0.59–3.82; p = 0.85)^12^ (Table 2), and CSM (3 studies; HR = 1.42, 95%CI: 0.98–2.05; p = 0.07; I^2^ = 0%)^10–12^ (Table 2) were considered as outcomes. In the adjusted estimates, the risk of recurrence was more significant for the outcome BCR (3 studies; HR = 1.42; 95%CI: 0.98–2.05; p = 0.07; I^2^ = 0%) than for distant metastasis (1 study; HR = 1.50; 95%CI: 0.59–3.82; p = 0.85), as shown in Fig. 3 (p for interaction = 0.74). Pooling together the indexes of recurrence, ENE+ status remained associated with a significantly higher risk of recurrence in four studies (HR = 1.41, 95%CI: 1.14–1.74; p = 0.001; I^2^ = 0%, Fig. 3)^11, 12, 14, 15^, which was unaffected by publication bias (adjusted estimate: HR = 1.32, 95%CI: 1.11–1.62, with two studies trimmed to the left of the mean). None of the outcomes analyzed indicated a high heterogeneity (as indicated by I^2^ ≥ 50%), so meta-regression and sensitivity analyses were not performed.

**Table 2:** Pooled risk ratio estimates for adjusted hazard ratios for overall and disease-free survival by presence or absence of extranodal extension.

Parameter	No. of studies	Hazard ratios (95%CI)	P-value	Heterogeneity	Egger’s bias test; p-value	Trim and fill [trimmed]
ACM	1	1.50 (0.59–3.82)	0.85	—	—	—
CSM	3	1.42 (0.98–2.05)	0.07	I^2^ = 0	0.49; 0.70	Unchanged
ROR	4	**1.41 (1.14–1.74)**	**0.001**	I^2^ = 0	1.15; 0.14	1.32 (1.11–1.62)^2^

Abbreviations: ENE: extranodal extension; CI: confidence interval; ACM: all-cause mortality; CSM: cancer-specific mortality; ROR: risk of recurrence.

Bold values are significant results with p-values < 0.05.

## Discussion

For this study we analyzed 6 observational studies involving 1,113 patients with N1 PCa, 658 of them ENE+, and 455 ENE−. Our results indicate that ENE is associated with a higher risk of recurrence (ROR) in PCa, and this association is particularly strong for the risk of BCR. Notably, the association was not just maintained, but even reinforced when HRs adjusted for potential confounders were considered. The robustness of our findings is confirmed by the fact that we did not find any significant heterogeneity or publication bias. At the same time, ENE was not associated with ACM or CSM in our meta-analysis. Judging from our results, ENE appears to be another factor capable of influencing the clinical history, and consequently also the quality of life of patients with PCa, but it does not seem to significantly affect their survival. So far, too few morphological features of nodal metastases have been identified as significant prognostic moderators, and that is why our results seem important. The prognostic predictor generally thought to be the most significant is nodal cancer volume, which has been described as the strongest indicator of systemic progression and CSM^7, 8^. The factors judged important for stratifying patient survival include the total metastatic tumor volume and the diameter of the largest metastasis^7, 8, 12, 26^. Metastatic tumor volume has been strongly associated with other important prognostic factors like the Gleason score and DNA ploidy, confirming this parameter’s close link with the biological behavior of metastasizing PCa^27^. Notably, ENE was not strictly correlated with metastatic tumor volume, and this may be one of the reasons for its limited influence on the global survival of PCa patients. At the same time, ENE is a classic example of local aggressiveness (with the tumor invading perinodal adipose tissue), and this can explain its association with ROR.

Another parameter that pathologists can document with ease and that correlates with prognosis in PCa patients is the number of positive lymph nodes^8^. A recent expert report suggested sub-stratifying N1 patients as: N1 if there is a single positive lymph node, and N2 if there are two or more. N1 could also be further divided according to the size of the tumor metastasis^27^. On the basis of our study, N1 patients could be subdivided by ENE status too, with ENE+ cases indicating a subgroup of N1 patients at greater ROR. Surprisingly, both the previous and the currently used staging system (the new AJCC cancer staging manual, recently released) only distinguishes between PCa patients with and without nodal involvement in a dichotomous way (N0 versus N1)^16, 28^. Future staging systems may consider the above-mentioned morphological features of lymph node metastases to better stratify patients and facilitate the identification of higher-risk patients who need to be followed up more closely, also on the basis of possible findings of future researches.

Another relevant implication emerging from this meta-analysis is about the sphere of surgical pathology, and gross sampling in particular. Standard sampling usually involves examining manually isolated, palpable lymph nodes^29^. Using this approach, a single lymph node may be oversampled and also counted more than once, since several different pieces can be obtained by the pathologist, especially in the case of large lymph nodes, while small metastatic lymph nodes may be overlooked. To avoid this problem, a recent report suggested using a technique that involves submitting all nodal and perinodal tissue, and examining palpable lymph nodes and the remaining tissue separately^30^. Consistently with this view, in the light of our finding that ENE has a certain prognostic importance in PCa, and the fact that it may be a very focal aspect in a metastatic lymph node, we would emphasize the importance of examining the whole of each lymph node, however large. Montironi *et al*. recently analyzed the potential clinical significance of the so-called large-format histology coupled with the total submission of nodal and perinodal tissue^31^, finding that this method improves the number of lymph nodes recovered and the detection of metastases. The approach has other advantages too, including a shorter time spent on sampling and fewer blocks needing to be cut and fewer slides to be examined^31^. Despite criticism relating to its higher costs, the superiority of such method is demonstrated by the fact that it enables nearly three times more lymph nodes to be identified than the standard sampling technique^31^. The use of fat-cleaning agents could be of help in this line, reducing the amount of tissue which has to be examined. On the grounds of these considerations, also regarding the increased opportunities in ENE detection, this technique may be taken into account by pathologists.

In our study the prognostic parameter most associated with the presence of ENE was BCR. Fleischmann *et al*. classified BCR as a PSA level >0.2 ng/ml. Hofer *et al*. indicated BCR as a postoperative increase in serum PSA to more than 0.4 ng/ml on two consecutive measurements. Passoni *et al*. defined BCR as two consecutive PSA readings >0.2 ng/ml. Irrespective of such small differences in the threshold considered, BCR seems to be significantly influenced by the presence of ENE, in terms of both risk ratios (RRs, Fig. 2), and hazard ratios (HRs, Fig. 3). Since BCR is considered an important prognostic parameter (also influencing PCa patients’ quality of life), we would emphasize the prognostic importance of ENE. ENE retains its prognostic significance for all types of recurrence (distant metastasis, BCR) (Figs 2 and 3), and it may be that, with a longer follow-up, the power of ENE to predict ACM and CSM may increase, and become statistically significant. Its impact on ROR is already important, however.

Another point of interest concerns adjuvant therapy. In a recent study on pancreatic cancer, patients with ENE seemed to have a better prognosis with the use of adjuvant chemoradiation, but not from chemotherapy alone^32^. This matter has to be further investigated because, if confirmed in PCa, such results could orient towards particular therapeutic options.

The standard definition of ENE is another important issue that warrants attention. Five out of the six studies considered here assessed ENE using a classical definition, such as the extension of tumor cells into the perinodal soft tissue^10, 12–15^. On the other hand, Cheng *et al*. considered metastatic deposits within adipose tissue as ENE as well, introducing a possible bias. It would be best to arrive at a standardized definition of ENE because of its possible importance in histopathological diagnostics. This parameter must be documented correctly in future studies, also because it may be an important prognostic moderator.

Whilst the results of this investigation appear as reliable, we must also consider some limitations. The first is represented by the small number of studies involved (which came as a surprise, considering the large body of literature on PCa). The sizable number of patients considered in each study (mean 186 patients/study), the studies’ high NOS scores (median = 8), and the limited heterogeneity of the results provide some guarantee of reliability, however. Further studies on ENE in PCa are nonetheless needed to clarify its prognostic potential. In addition, data on other comorbidities were not indicated in the studies analyzed, though they are known to play a significant part in the prognosis of patients with PCa.

In conclusion, our meta-analysis indicates that ENE is associated with the risk of BCR and of disease recurrence in general, even after adjusting for potential confounders. Notably, ENE is identified in a remarkable proportion of N1 PCa patients. However, we have to recognize that, at this moment, there are no different treatment options for patients with or without ENE, at the time that the initial pathology report is generated. Also on the basis of our work, this parameter may be taken into account by future studies about advanced PCa with nodal metastases. At the other hand, it is also true that now the follow-up will be the same for either type (ENE+ and ENE−) of patient. Concluding this part, if ENE becomes part of a standard CAP synoptic report, at this moment it should be only one of the “optional parameters”.

Further studies are needed, preferably with a longer follow-up, to shed more light on the potential role of this parameter in influencing PCa patients’ survival. Our results may become more relevant with the passage of time and the accumulation of additional data, and the development of new therapeutic treatments options for advanced prostate cancer. A final consideration regards the recent development of molecular techniques. Indeed, it has been indicated that pathology reports will be integrated with a specific molecular profile of a patient’s cancer^33–37^, but, before taking up such a proposal, all the prognostic value of all morphological aspects, such as ENE, have to be clarified.

## Methods

This systematic review has been performed following the Meta-Analysis Of Observational Studies in Epidemiology (MOOSE) guidelines^38^ and the Preferred Reporting Items for Systematic reviews and Meta-Analyses (PRISMA) statement^39^ (Supplementary Fugure 1).

### Data sources and literature search strategy

Two investigators (C.L., A.N.) independently conducted a literature search in PubMed and SCOPUS with no language restrictions, from the inception of the databases up until 30 June 2016, seeking prospective studies that compared any prognostic parameters (all-cause mortality, cancer-specific mortality and recurrent disease) in patients with a diagnosis of nodal positive PCa with or without extranodal disease (ENE+ vs. ENE−). In PubMed, controlled vocabulary terms and the following keywords were used: (“extracapsular” OR “pericapsular” OR “extranodal” OR “perilymphatic” OR “perinodal” OR “extra capsular” OR “peri capsular” OR “extra nodal” OR “peri lymphatic” OR “peri nodal” OR “extra-capsular” OR “peri-capsular” OR “extra-nodal” OR “peri-lymphatic” OR “peri-nodal”) AND (“prostat*”) AND (“mortality” OR “mortalities” OR “fatality” OR “fatalities” OR “death*” OR “survival” OR “prognosis” OR “hazard ratio” OR “HR” OR “relative risk” OR “RR” OR “progression” OR “recurrence”). The same search was conducted also in SCOPUS. Conference abstracts were also evaluated, and so were the reference lists of included articles, and those identified as relevant to the topic were hand-searched to identify any additional, potentially relevant articles. Any inconsistencies were solved by consensus.

### Study selection

For this meta-analysis, as inclusion criteria we considered: (1) prospective, observational cohort studies; (2) comparison of prognostic factors between ENE− vs. ENE+ cases; (3) a clear diagnosis of nodal-positive acinar prostate adenocarcinoma; (4) data concerning mortality or recurrent disease, considering not only local recurrences or distant metastases, but also biochemical recurrence (BCR).

As exclusion criteria we considered: (1) no ongoing cancer; (2) absence of data on prognostic indicator in the title/abstract; (3) comparisons between ENE + vs. N0 (no lymph node metastases); (4) a diagnosis of cancer histotypes other than acinar prostate adenocarcinoma; and (5) animal or *in vitro* studies.

### Data extraction

One author (M.S.) was involved in data extraction from the articles included in the analysis and a second author (C.L.) checked independently the resulting data. The information extracted regarded the authors, year and country of publication, exclusion criteria, number of patients with metastatic lymph nodes, patients’ age, type and mode of the recurrence of disease (if any), number of adjustments in survival analyses, and duration of follow-up. If we did not find some information on ENE or outcomes, we contacted the first and/or corresponding authors of the specific original articles to obtain unpublished data. In case two articles referred to the same cohort, the most recent study was considered.

### Outcomes

We considered as primary outcomes these parameters as follows: the number of deaths irrespective of their cause (all-cause mortality); the number of deaths due to cancer; and the number of recurrences in ENE+ vs. ENE− node positive PCa patients during the follow-up. We considered as secondary outcomes these parameters as follows: hazard ratios (HRs), adjusted for the maximum number of confounders available, for the above-mentioned parameters, taking ENE− patients for reference.

### Assessment of study quality

The Newcastle-Ottawa Scale (NOS) was used to judge the quality of the studies (http://www.ohri.ca/programs/clinical_epidemiology/oxford.htm), using a score of ≤5 (out of 9) as a indicator for a high risk of bias^40^.

### Data synthesis and statistical analysis

We performed the analyses using Comprehensive Meta-Analysis (CMA) 3 (http://www.meta-analysis.com). To test for normality in the case of continuous variables, the Shapiro-Wilk test was used. If normality was satisfied, we presented the variables as means ± standard deviations; if not, we considered medians and ranges.

In primary analyses, pooled risk ratios (RRs) and 95%CIs for all-cause mortality, cancer-specific mortality, and disease recurrence were calculated between ENE+ and ENE− cases applying DerSimonian-Laird random-effects models^41^. In secondary analyses, pooled HRs with 95%CIs adjusted for the maximum number of covariates available in the papers were also calculated to clarify whether potential confounders may affect the relationship between ENE status and outcomes. We assessed heterogeneity across studies with the Cochrane I^2^ metric and chi square statistics. In case of significant heterogeneity (p < 0.05)^42^, a series of meta-regression analyses by ENE status and each of the prognostic parameters considered will be conducted.

Publication bias was assessed by visual inspection of funnel plots and also using the Begg-Mazumdar Kendall tau^43^, and the Egger bias tests^44^. The trim-and-fill method was also applied for a final check about publication bias^44, 45^.

## Electronic supplementary material

Supplementary Information
