# Supplementary material for: Extranodal extension of lymph node metastasis influences recurrence in prostate cancer: a systematic review and meta-analysis
Source: Sci Rep. 2017 May 24;7:2374. doi: 10.1038/s41598-017-02577-4 (PMC5443831; doi:10.1038/s41598-017-02577-4)
Supplement: Supplementary file 1 — Supplementary Information [file 41598_2017_2577_MOESM1_ESM.pdf]

Supplementary Information of the paper entitled:

"Extranodal extension of lymph node metastasis influences recurrence in prostate cancer: a systematic review and meta-analysis".

Authors list: Claudio Luchini, Achim Fleischmann, Joost L. Boormans, Matteo Fassan, Alessia Nottegar, Paola Lucato, Brendon Stubbs, Marco Solmi, Antonio Porcaro, Nicola Veronese, Matteo Brunelli, Aldo Scarpa, Liang Cheng

Supplementary Figure 1. PRISMA diagram for this meta-analysis.

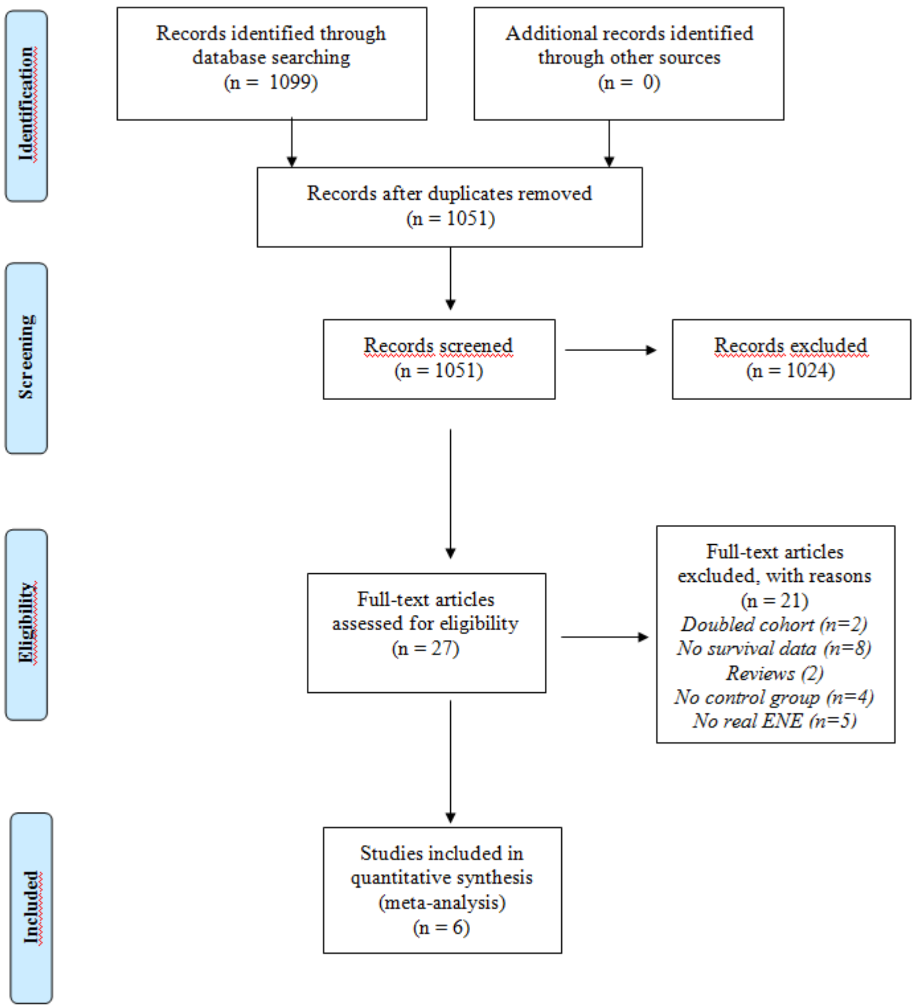

Supplementary Table 1. Characteristics of the studies, taking only N+ patients into account.

		N+ ENE+				N+ ENE-							
Study Author, Year (Country)	Exclusion criteria	Number of patients	Mean age ± SD	Gleason score	No. of positive nodes	Number of patients	Mean age ± SD	Gleason score	No. of positive nodes	ENE definition*	Number of adjustments (type)	Mean follow-up (months)	NOS
**Boormans, 2008 (Nether-lands)**	Preoperative treatment, no data regarding death	82	63±7	6: 1%, 7: 54%, 8: 28%, 9-10: 17%	3	63	63±7	6: 13%, 7: 48%, 8: 22%, 9-10: 17%	2	1	4 (Age, pN, Gleason score, adjuvant radio-therapy)	77.5	9
**Cheng, 2000 (USA)**	Preoperative treatment, cancer volume and/or ploidy analysis, and/or histological slides not available	126	66	NA	2	86	67	NA	1	2	0	73	9

**Fleischmann, 2008 (Switzer-land)**	Neoadjuvant therapy	71	64 (45-75)	6: 10%, 7: 43%, 8: 20%, 9-10: 27%	3	31	65 (51-72)	6: 10%, 7: 43%, 8: 20%, 9-10: 27%	1	1	1 (Diameter of largest metastasis)	92	9
**Griebling, 1997 (USA)****	Incomplete histological specimens, preoperative RT	33	NA	NA	NA	18	NA	NA	NA	1	0	40.2	7
**Hofer, 2006 (Germany)**	Preoperative hormonal ablation therapy	66	NA	NA	NA	53	NA	NA	NA	1	0	NA	7

**Passoni, 2013 (Europe and North America)*** **	Lack of accurate treatment regimen	280	NA	NA	NA	204	NA	NA	NA	1	8 (Age, preoperative PSA level, Gleason score, pT, positive surgical margins, >2 positive lymph nodes, positive lymph node diameter > 10 mm, adjuvant radio-therapy)	16.1	7
**All studies (means and percentages weighted with n values only if available*)***		**658**	**65±7**	**6: 6%, 7: 49%, 8: 24%, 9-10: 22%**	**Median=3 (range: 2-3)**	**455**	**64±8**	**6: 12%, 7: 46%, 8: 21%, 9-10: 22%**	**Median=1 (range: 1-2)**	**5 studies: definition 1; 1 study: definition 2.**	**Median =1 (range: 0-8)**	**Median =83 (range: 16-92)**	**Median=8 (range: 7-9)**

Abbreviations: NA: not applicable, or not assessed, or not specified, RT: radiotherapy; ENE: extranodal extension; NO: Newcastle-Ottawa Scale; pT: pathological T stage; pN: pathological N stage.

Notes:

* Methods of ENE assessment:

1. ENE defined as penetration of the tumor cells beyond the lymph node capsule into the perinodal fatty tissue;

2. ENE defined as cancer perforating the capsule and extending into the perinodal tissue; metastatic deposits within fat were also counted as ENE.

**In this study, the Authors divide patients into three categories: ENE+, ENE-, and ENE equivocal; for the purposes of our analysis, the ENE equivocal cases were disregarded.

*** Data at 36 months of follow-up were used in this study (figure 3a), the longest available in the manuscript.

Supplementary Table 2. Newcastle-Ottawa Scale (NOS) Table: Methodological quality of cohort studies included in the meta-analysis*

First author, publication year	Representative-ness of the cohort (ENE+ vs ENE-)	Selection of the un-exposed cohort	Ascertain-ment of exposure[†]	Outcome of interest not present at start of study[††]	Control for important factor or additional factor[†††]	Assess-ment of outcome	Follow-up long enough for outcomes to occur[††††]	Adequacy of follow-up of cohorts	Total quali-ty scores
**Boormans, 2008**	*	*	*	*	**	*	*	*	9
**Cheng, 2000**	*	*	*	*	**	*	*	*	9
**Fleischmann, 2008**	*	*	*	*	**	*	*	*	9
**Griebling, 1997**	*	*	*	*	*	*	-	*	7
**Hofer, 2006**	*	*	*	*	*	*	-	*	7
**Passoni, 2013**	*	*	*	*	*	*	-	*	7

* A study could be awarded a maximum of one star for each item, except for "Control for important factor or additional factor". The definition/explanation of each column of the Newcastle-Ottawa Scale is available at: http://www.ohri.ca/programs/clinical_epidemiology/oxford.htm.

[†] For this index, one star was given if in the Methods section ENE was clearly assessed at histology and its definition was clearly stated.

[††] Since the outcome of interest was mortality, for quality assessment purposes we took whether disease-specific survival or recurrence rate (including biochemical recurrence) were assessed as the outcome of interest.

[†††] A maximum of 2 stars could be awarded for this item. Studies that controlled their survival analyses for at least two confounders received one star, and those presenting data on ENE considering only patients who had undergone standardized bilateral pelvic lymphadenectomy were awarded an extra star.

[††††] A cohort study with a mean/median follow-up ≥5 y (60 months) was awarded one star; no star was awarded if the mean follow-up was not clearly indicated or deducible.
